# Complete Genome Sequence of Thalassotalea euphylliae Strain H2

**DOI:** 10.1128/MRA.01608-18

**Published:** 2019-01-24

**Authors:** Stephen Summers, Marnie L. Freckelton, Brian T. Nedved, Scott A. Rice, Michael G. Hadfield

**Affiliations:** a Singapore Centre for Environmental Life Sciences Engineering, Nanyang Technological University, Singapore; b Kewalo Marine Laboratory, University of Hawai’i, Honolulu, Hawaii, USA; c The School of Biological Sciences, Nanyang Technological University, Singapore; Broad Institute of MIT and Harvard University

## Abstract

A bacterial isolate of Thalassotalea euphylliae H2 was collected from the coral Montipora capitata. MinION long reads were employed for scaffolding and complemented with short-read MiSeq sequences to permit complete genome assembly. The genome is approximately 4.36 Mb long, with 3,669 protein-coding genes, 92 tRNAs, and 21 rRNAs.

## Abstract (precis)

A bacterial isolate of Thalassotalea euphylliae H2 was collected from the coral Montipora capitata. MinION long reads were employed for scaffolding and complemented with short-read MiSeq sequences to permit complete genome assembly.

## ANNOUNCEMENT

The bacterium Thalassotalea euphylliae is a Gram-negative rod-shaped bacterium isolated from the coral Euphylliae glabrescens (1). This bacterium is motile, having a single polar flagellum, and it forms a pale-yellow colony. Other members of the species Thalassotalea euphylliae have also been isolated and their genomes reported (2). This bacterium has been associated with a range of marine environments, such as oyster tissues (3), tidal flats (4), marine sediments (5), and seawater (6).

Here, we present the genome of Thalassotalea euphylliae H2, isolated from the mucus of the coral Montipora capitata. The coral was sampled on the reef adjacent to the Hawaii Institute of Marine Biology (University of Hawai’i) in Kane’ohe Bay, O’ahu, Hawaii, in 2010 (7). The bacterium was isolated from the surface of coral branches by streak plating on half-strength seawater-tryptone agar (8). The culture is retained at −80°C at the Kewalo Marine Laboratory (University of Hawai’i, Honolulu, HI).

The isolate was grown overnight at 28°C in a half-strength seawater-tryptone broth (8), and the cells were collected by centrifugation at 4,000 × *g* for 5 min. Total nucleic acids were extracted from the cell pellets using a xanthogenate-based extraction technique (9). Following extraction, 300-bp paired-end sequencing was carried out on the Illumina MiSeq platform after library preparation using a TruSeq Nano DNA kit, which yielded 2,534,742 reads. Illumina MiSeq sequencing adapters were removed using the Trimmomatic package (version 0.36), and quality control (QC) was performed using FastQC (version 0.11.7). Assembly was assisted by the use of long-read sequencing as a scaffold. This was performed on a Nanopore MinION genome sequencer using a rapid sequencer kit (SQK-RAD004) and a R9.4 flow cell. This resulted in 536,912 reads of up to 131,642 bp in length (median, 5,874 bp).

The long reads were base called using the Albacore package version 2.3.1, as supplied by Nanopore, using default settings, and adapters were removed using Porechop version 0.2.3 (verbosity level 3). The *de novo* assembly was conducted using the Unicycler package version 0.4.4 (10) using long reads as a scaffold to enhance MiSeq assembly of the genome. Polishing and rotation were performed using the default settings in Racon version 1.3.1 (11).

The final polished circular chromosomal contig was 4,357,276 bp (174-fold coverage, 43.0% GC content). Species identification was achieved by BLASTn comparison of the NCBI 16S rRNA gene database and showed a 98% match to T. euphylliae strain Eup-16 (NCBI RefSeq accession number NR_153727).

Genome annotation was completed with the NCBI Prokaryotic Genome Annotation Pipeline (PGAP) version 4.2 (12). A total of 3,808 genes were identified, consisting of 3,669 protein-coding genes and 115 RNAs (92 tRNAs, 4 noncoding RNAs, 7 5S rRNA subunits, and 6 copies each of the 16S and 23S rRNAs). Of these, 31 genes were identified as related to membrane function and maintenance. Using the functional annotation services provided by the Rapid Annotations using Subsystems Technology (RAST [13]) servers, we identified 3,897 DNA-coding sequences within 151 subsystems.

### Data availability.

The genome sequence of T. euphylliae H2 has been deposited in GenBank under accession number QUOV00000000. Raw sequencing reads are available in the NCBI Sequence Read Archive (accession numbers SRR8069230 and SRR8069231).
